# Anomalous Nonlinear Optical Effects by Intensity-Dependent Phase-Variation Compensation in Photonic Crystals Containing Hyperbolic Metamaterials

**DOI:** 10.3390/nano15120903

**Published:** 2025-06-11

**Authors:** Xiangting Yu, Haoyuan Qin, Junyang Li, Hong Chen, Xudong Li, Fen Liu, Tongbiao Wang, Guang Lu, Guiqiang Du

**Affiliations:** 1 School of Space Science and Physics, Shandong University, Weihai 264209, China; 202000830074@mail.sdu.edu.cn (X.Y.); 202100830071@mail.sdu.edu.cn (H.Q.); 202117744@mail.sdu.edu.cn (J.L.); 202121024@mail.sdu.edu.cn (H.C.); xvdongli@mail.sdu.edu.cn (X.L.); liufen210@sdu.edu.cn (F.L.); 2 School of Physics and Materials Science & Jiangxi Provincial Key Laboratory of Photodetectors, Nanchang University, Nanchang 330031, China; tbwang@ncu.edu.cn; 3 Weihai Research Institute of Industrial Technology of Shandong University, Shandong University, Weihai 264209, China

**Keywords:** photonic crystals, metamaterials, hyperbolic metamaterials, nonlinear optical effects

## Abstract

We theoretically investigated two types of nonlinear optical effects of photonic band edges (PBEs) in photonic crystals containing hyperbolic metamaterial (HMM) based on the intensity-dependent phase-variation compensation, where the HMM is composed of alternating the noble metal Ag with large-nonlinear-coefficient and dielectric material. Considering nonlinear conditions, the local field strength variation in nonlinear materials with the increase in the incident angle will lead to the movement of the PBE, resulting in two anomalous optical nonlinear effects. When the PBE is angle-independent under the linear condition, the PCs have angle-sensitive optical bistability and the critical threshold intensity always increases. However, if the PBE is designed to have angle dependence under linear conditions, the optical bistability in the PC can be angle-independent, and the critical threshold intensity is angle-independent over a wide range. This research provides important reference values for manufacturing direction-selectable devices that utilize different kinds of nonlinear optical effects.

## 1. Introduction

Nonlinear optical effects have been investigated in several disciplines, including physics, materials, and information technology [1,2,3,4,5]. Nonlinear optical effect regulation is determined by nonlinear optical coefficients of the subject materials. However, ideal nonlinear optical properties are challenging, as the bulk material adjustment parameters are limited. For example, some noble metals exhibit higher nonlinear coefficients than those of typical dielectric materials. However, it is difficult for EM fields to enter the interior of bulk metal materials, so they have high critical threshold intensities for nonlinear optical effects [6,7,8]. Since photonic crystals (PCs) were proposed [9], they have attracted much attention due to the fact that significant nonlinear optical effects with low threshold intensities can be realized by inserting nonlinear optical materials into PCs [6,7,8,10,11,12,13,14,15,16,17,18]. For example, metal–dielectric PCs, composed of noble metal layers and dielectric layers, can greatly enhance nonlinear optical effects [6,7,8,12,13,14,19]. In addition, by combining a thick metallic film with high-nonlinearity and all-dielectric PCs to form a photonic heterostructure, optical bistability with very low threshold intensities and high-efficiency photodiodes can be achieved based on localized interface modes [12,20]. However, the photonic band edges (PBEs) of conventional all-dielectric and metal–dielectric PCs are angle-sensitive [21,22], which limits their development and application in engineering omnidirectional nonlinear optical devices.

Since 2001, the negative refractive index has been experimentally demonstrated in metamaterials [23]; these kinds of subwavelength micro/nanostructures with arbitrary permittivity or permeability have attracted wide attention [6,23,24,25,26]. Hyperbolic metamaterial (HMM) with effective hyperbolic permittivity or permeability dispersion curves possesses an outstanding ability to modulate the propagation of electromagnetic waves [27,28,29,30,31,32]. According to the effective medium theory, electric HMM can be realized using subwavelength metal–dielectric multilayers and the propagating phase in HMM is opposite to that in dielectric materials for TM polarization [33,34,35]. Based on the phase-variation compensation effect [31,34,35,36], three PCs composed of alternating an HMM and a dielectric material are produced, employing a novel PBG, including angle-insensitive and anomalous red-shifted band edges [30,33,34,35,36], enabling the development of high-performance omnidirectional mirrors, perfect optical absorbers, omnidirectional polarization beam splitters, and other optical devices. However, if the nonlinear optical coefficient of the metal in the HMM is considered, the effective permittivity of the HMM will depend on the light intensity. Therefore, the phase-variation compensation between a nonlinear HMM and a dielectric material will be intensity-dependent. Based on the intensity-dependent phase-variation compensation mechanism, we systematically study two kinds of nonlinear optical effects of the PBE in PCs containing HMM.

## 2. Theoretical Model

The nonlinear transfer matrix method [37] is used to calculate the linear and nonlinear optical transmission characteristics of PCs. For non-magnetic materials (μ=1), the tangential components of the electric and magnetic fields passing through the *i*-th layer with a thickness of $d_{i}$ and a refractive index of $n_{i}$ are correlated by the characteristic matrix

(1) $$M_{i}=\left[\begin{array}{cc} \cos\delta_{i} & i{\eta_{i}}^{-1}\sin\delta_{i}\\ i\eta_{i}\sin\delta_{i} & \cos\delta_{i} \end{array}\right]$$

where

$$\delta_{i}=\frac{2\pi }{\lambda }n_{i}d_{i}\cos\theta ,$$

$$\eta_{i}=n_{i}/\cos\theta ,$$

for TM polarization, and θ is the angle of incidence. The transfer matrix for the multilayer can be obtained by multiplying together the entire characteristic matrix:

(2) $$\left[\begin{array}{c} B\\ C \end{array}\right]=\prod_{i=1}^{N}M_{i}\left[\begin{array}{c} 1\\ \eta_{i+1} \end{array}\right],$$

The transmittance of PCs is given by

(3) $$\;T=\frac{4\eta_{0}\eta_{i+1}}{(\eta_{0}B+C){\left(\eta_{0}B+C\right)}^{*}},$$

where $\eta_{0}$ and $\eta_{i+1}$ are the admittance of input and output materials. If nonlinear materials are introduced into PCs, the nonlinear layer is divided into many sub-layers, in each of which the refractive index can be regarded as constant. The input field is defined as plane waves propagating in a layered structure from free space, which is described as follows:

(4) $$\;E=E^{+}e^{ikz}+E^{-}e^{-ikz},$$

where k is the propagation wavenumber in the layer. The wave amplitudes in two adjacent layers *j* and *j* + 1 can be related by boundary continuity conditions, which can be written in matrix form:

(5) $$\left[\begin{array}{c} E_{j+1}^{+}\\ E_{j+1}^{-} \end{array}\right]=\frac{1}{2}\left[\begin{array}{cc} 1+\frac{\eta_{j}}{\eta_{j+1}} & 1-\frac{\eta_{j}}{\eta_{j+1}}\\ 1-\frac{\eta_{j}}{\eta_{j+1}} & 1+\frac{\eta_{j}}{\eta_{j+1}} \end{array}\right]\left[\begin{array}{cc} e^{i\delta_{j}} & 0\\ 0 & e^{-i\delta_{j}} \end{array}\right]\left[\begin{array}{c} E_{j}^{+}\\ E_{j}^{-} \end{array}\right],$$

and the nonlinear dielectric constant is given by

(6) $$\varepsilon^{NL}=\varepsilon^{L}+\varepsilon_{0}\chi_{3}{\left|E\right|}^{2},$$

where $\chi_{3}$ is the third-order susceptibility, $\varepsilon^{L}$ is the linear dielectric constant, and the nonlinear dielectric constant of sub layer *j* + 1 can be derived from boundary condition (5):

(7) $$\;\varepsilon_{j+1}^{NL}=\varepsilon_{j+1}^{L}+\varepsilon_{0}\chi_{3}{\left|E_{j}^{+}e^{i\delta_{j}}+E_{j}^{-}e^{-i\delta_{j}}\right|}^{2}.$$

In this way, the refractive index of the sub-layers can be calculated layer by layer.

## 3. Nonlinear Optical Effects in Metal–Dielectric Photonic Crystals

**Figure 1:**
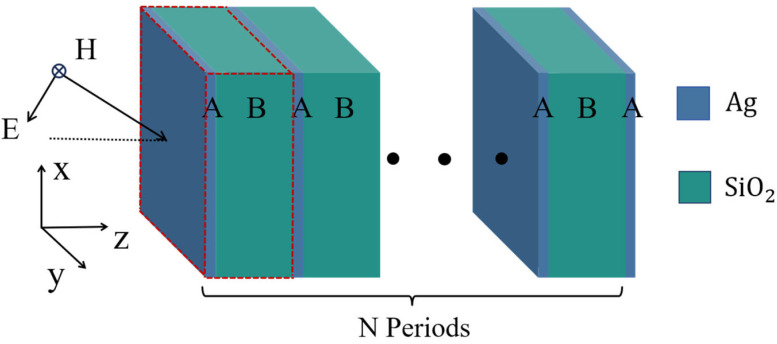
The truncated metal–dielectric PCs ware designed as (AB)^N^A. A, B, and N represent Ag, SiO_2_, and 6, respectively. *d*_A_ = 23.0 nm and *d*_B_ = 140.3 nm.

Firstly, we consider a conventional metal–dielectric photonic crystal and analyze its PBE and nonlinear properties. The conventional PCs (AB)^N^A were composed of metal A and dielectric materials B, as shown in Figure 1. A, B, and N represent the nonlinear optical material (Ag), SiO_2,_ and periodic number, respectively. The linear dielectric constant of Ag in the HMM was obtained using the Drude model [12,38]:

(8) $$\varepsilon_{Ag}^{L}=1.0-\frac{\omega_{p}^{2}}{\omega^{2}+i\gamma \omega }$$

where $\hbar \omega_{p}=7.2\;\mathrm{eV},\hbar \gamma =0.05\;\mathrm{eV}$, and γ is the damping frequency, which determines the loss of Ag. The relative permeability of Ag is supposed as $\mu_{Ag}=1$, $\chi_{3}=2.4\times 10^{-9}esu$ [4,12]. The period number is 6 and the thicknesses of Ag and SiO_2_ are chosen to be 23.0 nm and 140.3 nm, respectively, as shown in Figure 1.

**Figure 2:**
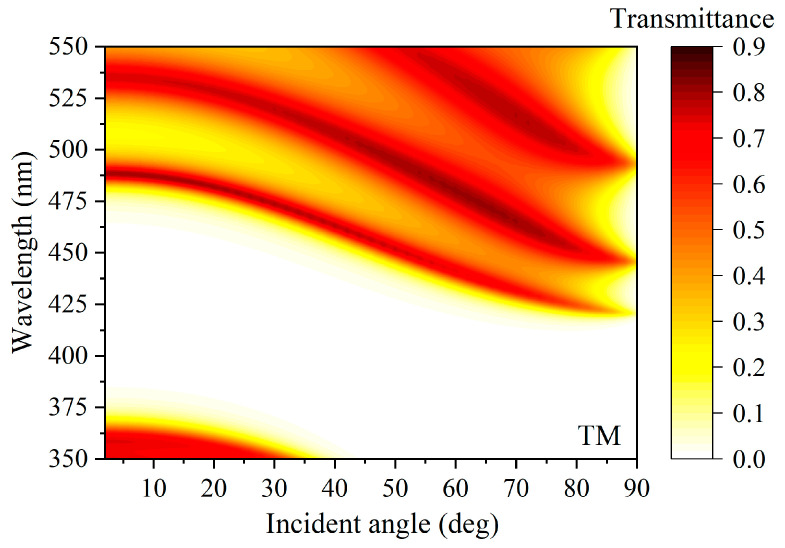
Transmission spectra of (AB)^N^A with exhibited blue-shifted PBG for oblique incidence of TM wave at 10.0 GW/cm^2^ incident electric field intensity.

We assumed the incident and exit media are both air and investigated the PBE variation of (AB)^N^ with the incident angle for the TM wave at a certain incident electric field intensity of 10.0 GW/cm^2^, as shown in Figure 2. The upper and lower band edges of the photonic bandgap gradually move to the short wavelength with the increase in incident angle, which can be called as blue-shifted band edges.

**Figure 3:**
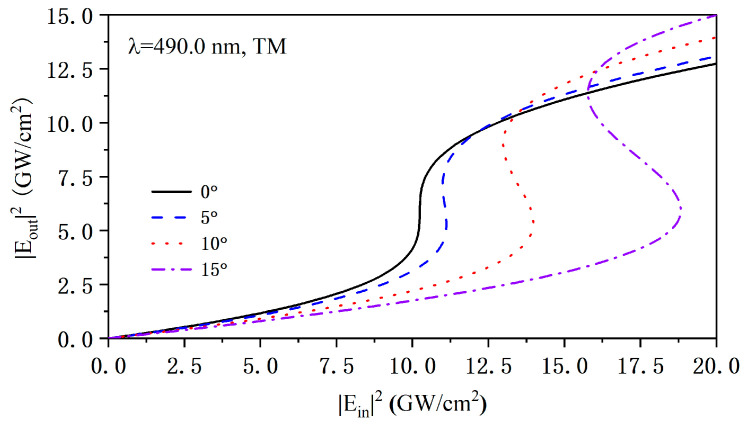
Optical bistability with incident angles at incident wavelength of 490.0 nm.

Moreover, we investigated the relationship between the nonlinear optical effects and the incident angle of the TM wave near the upper band edge of the PCs by following the nonlinear transfer matrix method [37]. As shown in Figure 3, we chose an incident wavelength of 490.0 nm and simulated the optical nonlinear transmission characteristics of truncated PCs (AB)^N^A under TM polarization at incident angles of 0°, 5°, 10°, and 15°. It is found that the critical threshold intensity of the optical bistability becomes larger rapidly with the increase in the incident angle. The localized electronic field at the band edge wavelength is higher than that away from band edge in the PCs, which results in enhanced nonlinear optical effects so that the critical threshold intensity is the lowest at normal incidence. Though the PBEs of PCs are affected by the light intensity, the band edge wavelength at the normal incidence still gradually moves away from the band edge of oblique incidence as the incident angle increases, which induces a lower localized electronic field within the PCs. As a result, the critical threshold intensity of optical bistability near the long-wavelength band edge gradually increases. Therefore, the angle-sensitive band edge is an important factor in angle-sensitive nonlinear optical effects.

## 4. Anomalous Nonlinear Optical Effects in Photonic Crystals Containing Hyperbolic Metamaterials

**Figure 4:**
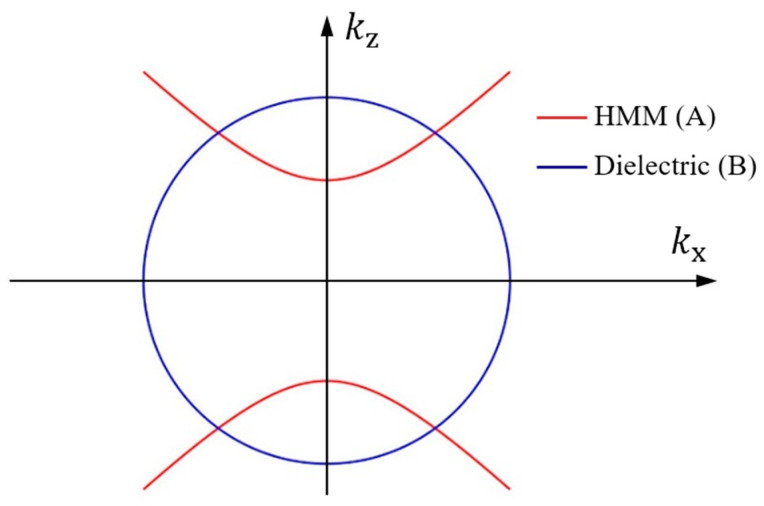
Iso-frequency curves of HMM (A) and dielectric material (B) for TM waves.

In recent years, many studies have shown that when HMM is introduced in PCs, such micro/nanostructures exhibit an angle-insensitive PBE based on phase-variation compensation [30,33]. We construct new PCs containing nonlinear HMM, which are denoted as (AB)^N^, wherein A, B, and N represent the HMM, the dielectric material, and the periodic number, respectively. The dielectric material is selected to be TiO_2_ and its refractive index is $n_{B}=$ 2.33 [12]. The iso-frequency curves of layers A and B are shown in Figure 4, where the special wavevector dispersion of HMMs provides a new method to engineer the PBE. According to the phase-variation compensation [30], the conditions to achieve an angle-insensitive PBG using PCs containing HMM can be evolved as follows:

(9) $${\left.\left(d_{A}\frac{\partial k_{Az}}{\partial k_{x}}+d_{B}\frac{\partial k_{Bz}}{\partial k_{x}}\right)\right|}_{\lambda_{Brg}}=0,$$

where $d_{A}$ and $d_{B}$ is the thickness of the HMM and dielectric, $k_{Az}$ and $k_{Bz}$ are the z component of the wave vector in the HMM and dielectric, and $k_{x}$ is the x component of the wave vector. It can be found that $k_{Az}$ in layer A increases but $k_{Bz}$ in layer B decreases as $k_{x}$ increases. Therefore, the phase variations were compensated in each unit cell AB of the PCs (AB)^N^. This altered the PBE shifting behavior of the PCs upon incident angle increment, so it can be used to construct PCs with a zero-shifted PBE with increasing angle [30,33,36]. The above discussion does not consider optical nonlinear effects. If optical nonlinear materials are introduced into HMM, the phase-variation compensation between the HMM and the dielectric material will depend on the light intensity since the dielectric constant of nonlinear material is a function of the local field strength, as shown in Equation (6). When the incident angle is changed, the change in local electric field strength will alter the refractive index of the nonlinear layer, which shifts the PBEs of the PCs [39]. When the local field strength increases, the refractive index increases, which induces a red-shifted PBE. If the local field strength decreases, the refractive index decreases, which induces a blue-shifted PBE. Therefore, when the nonlinear materials are introduced in PCs, the linear angle-insensitive PBE will change to be angle-dependent because the light intensity changes the condition of the phase-variation compensation. Based on the intensity-dependent phase-variation compensation, angle-insensitive PBEs in PCs containing linear HMM generally result in angle-sensitive nonlinear optical effects since the PBE in PCs containing nonlinear HMM will be red-shifted or blue-shifted.

According to the angle sensitivity of the PBE and threshold intensity under linear and nonlinear conditions, anomalous optical nonlinear effects can be divided into two types: (I) angle-insensitive PBEs under linear conditions exhibit angle sensitivity to threshold intensity under nonlinear conditions, and (II) angle-sensitive PBEs under linear conditions can be angle-independent under nonlinear conditions.

**Figure 5:**
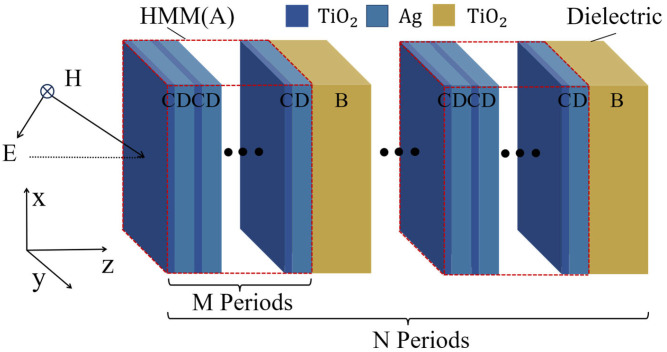
The PCs containing HMM are denoted as (AB)^N^. A denotes the HMM layer (CD)^M^. B and C denote TiO_2_, while D denotes Ag.

Firstly, we use a zero-shifted PBE under linear conditions to illustrate type (I). A structure based on phase-variation compensation is designed and its nonlinear transmission characteristics are analyzed, where the HMM (A) is composed of subwavelength metal–dielectric periodic multilayer films (CD)^M^, wherein C, D, and M denote TiO_2_, the noble metal Ag (exhibiting a large optical nonlinear coefficient), and periodic number, respectively. Therefore, the new PCs are denoted as [(CD)^M^B]^N^, as shown in Figure 5. An angle-independent PBE under linear conditions can be observed when the phase variation is compensated based on Equation (9). Firstly, we determine the geometric parameters of the HMM; according to the effective medium theory (EMT), the effective medium permittivity tensor in two directions can be written as [29] follows:

(10) $$\begin{array}{c} \varepsilon_{Ax}=f\varepsilon_{C}+\left(1-f\right)\varepsilon_{D}\\ \frac{1}{\varepsilon_{Az}}=f\frac{1}{\varepsilon_{C}}+\left(1-f\right)\frac{1}{\varepsilon_{D}} \end{array},$$

where $f=d_{C}{/(d}_{C}+d_{D})$ represents the dielectric-layer filling ratio. We set f=0.50, select $\lambda_{Brg}$ to be 344.8 nm, and finally obtain the thicknesses of the HMM and dielectric layers as $d_{HMM}=112.0\;\mathrm{nm}$ and $d_{B}=21.2\;\mathrm{nm}$, so this PC is angle-insensitive under linear conditions. The period number of HMM multilayer films is selected to be M = 16, and the period number of PCs is N = 6, so the PCs containing HMM are denoted by [(CD)^16^B]^6^, where $d_{C}={fd}_{HMM}/16=3.5\;\mathrm{nm}$ and $d_{D}={\left(1-f\right)d}_{HMM}/16=3.5\;\mathrm{nm}$.

**Figure 6:**
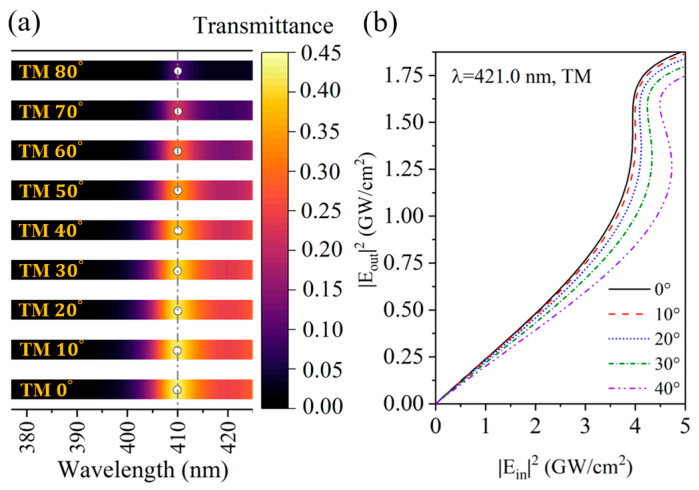
(**a**) Variation in band edge wavelength with incident angle under TM polarization under linear conditions; (**b**) optical bistability with incident angles at incident wavelength of 421.0 nm.

We simulated the transmission spectrum of the structure under linear conditions and its nonlinear transmission characteristics, as shown in Figure 6a,b. It can be found that the PC has a zero-shifted PBE under linear conditions but it has angle-sensitive optical bistability since the phase-variation compensation condition is changed by the light intensity. As the angle increases, the red-shifted PBE causes the fixed incident wavelength to enter the bandgap, which results in an increase in the critical threshold intensity. Therefore, regarding the angle-sensitive nonlinear transmission characteristics of the PBE, whether it is blue-shifted into the passband or red-shifted into the bandgap, the critical threshold intensity is always enhanced with the increase in the incident angle.

Secondly, we use an angle-sensitive PBE under linear conditions to illustrate type (II); the new PCs are designed under linear conditions. Similarly, we set f=0.26, select ${\lambda^{\prime }}_{Brg}$ to be 275.0 nm, and obtain the thicknesses of the HMM and dielectric layers as $d_{HMM}^{\prime }=76.0\;\mathrm{nm}$ and $d_{B}^{\prime }=41.0\;\mathrm{nm}$, so this PC is angle-insensitive under linear conditions. The period number of HMM multilayer films is selected to be M = 4, and the period number of the PCs is N = 3, so the PCs containing HMM are denoted by [(CD)^4^B]^3^, where C and D denote TiO_2_ and Ag, and $d_{C}^{\prime }=fd_{HMM}^{\prime }/4=5.0\;\mathrm{nm}$ and $d_{D}^{\prime }=(1-f)d_{HMM}^{\prime }/4=14.0\;\mathrm{nm}$. However, the PBG of the PC exhibits a small red-shift caused by an increase in local electric field strength under nonlinear conditions. Therefore, we need a small blue-shifted PBG of linear PCs to cancel out the effect of local electric field strength so that the thickness of dielectric is increased with $d_{B}^{\prime }=49.0\;\mathrm{nm}$.

**Figure 7:**
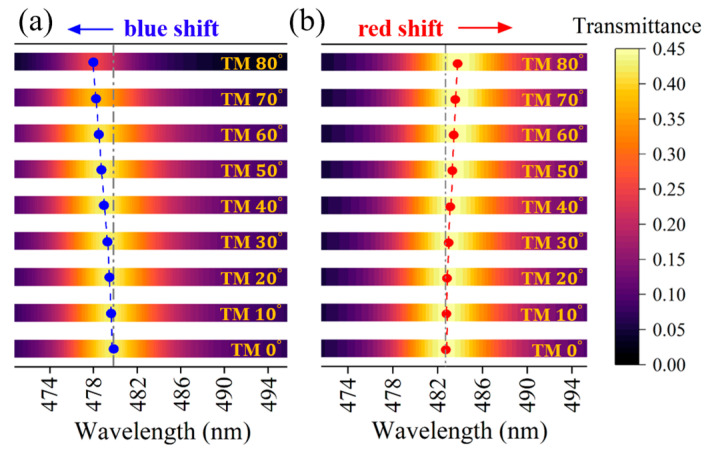
(**a**) Variation in band edge wavelength with incident angle under TM polarization under linear conditions; (**b**) variation in band edge wavelength with local electric field intensity.

**Figure 8:**
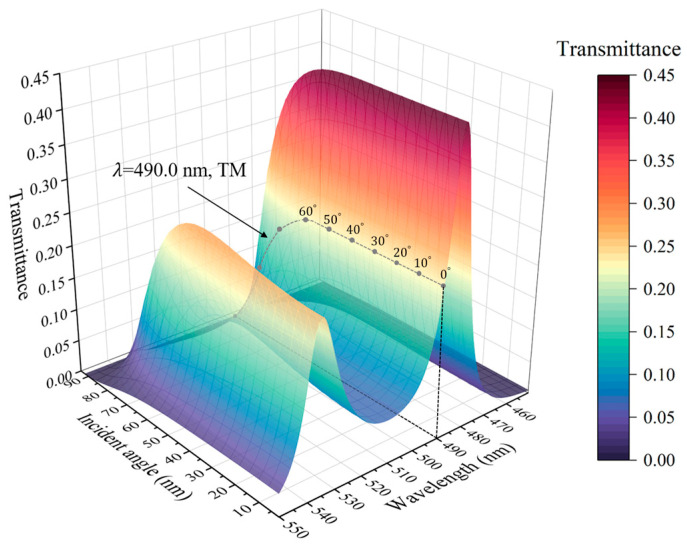
Transmission spectra depicting the angle-insensitive band gap of the PCs [(CD)^4^B]^3^ with oblique incidence of TM waves and an incident intensity of 1.5 GW/cm^2^.

**Figure 9:**
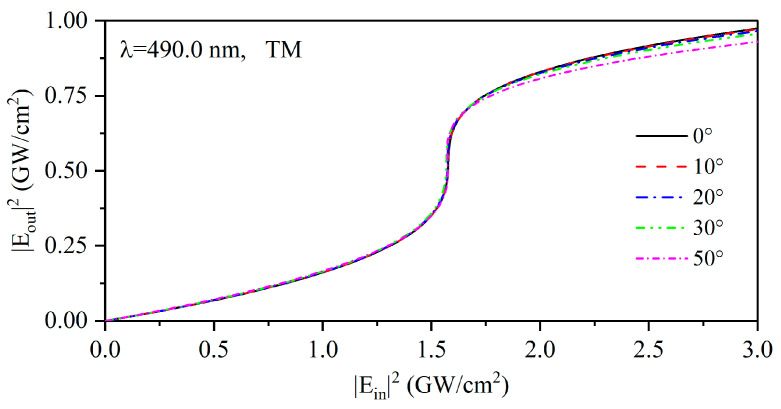
Optical bistability at a wavelength 490.0 nm for different incident angles of 0°, 10°, 20°, 30°, and 50°.

Considering the independent effect of the incident angle and local field strength, we simulated the transmission spectra of the PCs (AB)^3^ containing HMM at different incident angles under linear conditions, which is shown in Figure 7a, as well as the transmission spectra with different local field strengths under nonlinear conditions, as given in Figure 7b. It can be found that the transmission characteristic of the structure is angle-sensitive under linear conditions. However, when the nonlinear condition is considered, the transmission spectrum of PCs (AB)^3^ containing a nonlinear HMM at an incident electric field strength of 1.5 GW/cm^2^ is shown in Figure 8. It can be found that an angle-independent PBE emerges when the influences of the incidence angle and the local field strength are considered simultaneously, and the angle-independent transmittance is realized by the PCs at wavelengths of 490.0 nm from 0° to 50°. The change in angle sensitivity arises from the red-shift of the PBE due to the change in the local field strength as the angle increases, which gives the nonlinear response of the PCs [(CD)^4^B]^3^. The output electric field intensity ${\left|E\right|}_{out}^{2}$ for the PCs was calculated as a function of the incident electric intensity ${\left|E\right|}_{in}^{2}$, as shown in Figure 9. The optical bistable critical wavelength of this structure is 490.0 nm, at which the nonlinear transmission curve of the structure almost completely overlaps from 0° to 50°. The optical bistable threshold intensity remains about 1.5 GW/cm^2^, indicating that PCs containing HMM are angle-insensitive in terms of nonlinear effects. Such PCs containing HMM can be engineered to develop novel angle-insensitive nonlinear optical devices.

## 5. Conclusions

In summary, we investigated the intensity-dependent phase-variation compensation and two types of anomalous optical nonlinear effects in PCs containing nonlinear HMM. This study reveals a more comprehensive understanding of nonlinear optical effects in PCs containing HMM. The critical threshold intensity of nonlinear optical effect always increases as the angle increases, whether the PBE is blue-shifted or red-shifted. By designing the PBE with angle dependence under linear conditions, angle-independent nonlinear critical threshold strength can be achieved, which improves the robustness of optical bistability in practical applications. By utilizing the bistability property of the optical nonlinear effect, these kinds of micro-nanostructures are able to realize fast all-optical switches of optical signals, which has a great potential in the fields of optical logic, all-optical signal processing, etc. Highly integrated optical bistable devices are expected to achieve large-scale stable parallel processing of optical signals, which provides practical implementation solutions for future all-optical signal processors and all-optical computing chips.

## Data Availability

The data cannot be made publicly available upon publication because no suitable repository exists for hosting data in this field of study. The data that support the findings of this study are available upon reasonable request from the authors.
